# Optimal Lesion Size Index for Pulmonary Vein Isolation in High-Power Radiofrequency Catheter Ablation of Atrial Fibrillation

**DOI:** 10.3389/fcvm.2022.869254

**Published:** 2022-04-07

**Authors:** Chi Cai, Jing Wang, Hong-Xia Niu, Jian-Min Chu, Wei Hua, Shu Zhang, Yan Yao

**Affiliations:** Cardiac Arrhythmia Center, Fuwai Hospital, National Center for Cardiovascular Diseases, Chinese Academy of Medical Sciences, Peking Union Medical College, Beijing, China

**Keywords:** atrial fibrillation, radiofrequency, catheter ablation, pulmonary vein isolation, high-power, lesion size index, conduction gap

## Abstract

**Background:**

Although both high-power (HP) ablation and lesion size index (LSI) are novel approaches to make effective lesions during pulmonary vein isolation (PVI) for atrial fibrillation (AF), the optimal LSI in HP ablation for PVI is still unclear. Our study sought to explore the association between LSI and acute conduction gap formation and investigate the optimal LSI in HP ablation for PVI.

**Methods:**

A total of 105 consecutive patients with AF who underwent HP ablation guided by LSI (LSI-guided HP) for PVI in our institute between June 2019 and July 2020 were retrospectively enrolled. Each ipsilateral PV circle was subdivided into four segments, and ablation power was set to 50 W with target LSI values at 5.0 and 4.0 for anterior and posterior walls, respectively. We compared the LSI values with and without acute conduction gaps after the initial first-pass PVI.

**Results:**

PVI was achieved in all patients, and the incidence of first-pass PVI was 78.1% (82/105). A total of 6,842 lesion sites were analyzed, and the acute conduction gaps were observed in 23 patients (21.9%) with 45 (0.7%) lesion points. The gap formation was significantly associated with lower LSI (3.9 ± 0.4 vs. 4.6 ± 0.4, *p* < 0.001), lower force-time integral (82.6 ± 24.6 vs. 120.9 ± 40.4 gs, *p* < 0.001), lower mean contact force (5.7 ± 2.4 vs. 8.5 ± 2.8 g, *p* < 0.001), shorter ablation duration (10.5 ± 3.6 vs. 15.4 ± 6.4 s, *p* < 0.001), lower mean temperature (34.4 ± 1.4 vs. 35.6 ± 2.6°C, *p* < 0.001), and longer interlesion distance (4.4 ± 0.3 vs. 4.3 ± 0.4 mm, p = 0.031). As per the receiver operating characteristic analysis, the LSI had the highest predictive value for gap formation in all PVs segments, with a cutoff of 4.35 for effective ablation (sensitivity 80.0%; specificity 75.4%, areas under the curve: 0.87). The LSI of 4.55 and 3.95 had the highest predictive value for gap formation for the anterior and posterior segments of PVs, respectively.

**Conclusion:**

Using LSI-guided HP ablation for PVI, more than 4.35 of LSI for all PVs segments showed the best predictive value to avoid gap formation for achieving effective first-pass PVI. The LSI of 4.55 for the anterior wall and 3.95 for the posterior wall were the best cutoff values for predicting gap formation, respectively.

## Introduction

Radiofrequency (RF) ablation for pulmonary vein isolation (PVI) has become the standard treatment for patients with atrial fibrillation (AF) (1). Recent studies have shown that high-power (HP) ablation has been shown to be feasible and effective in achieving a high rate of PVI and reducing procedure complications (2–6). Previous *in vivo* and *ex vivo* studies have demonstrated that, in contrast to conventional low-power (LP) ablation, HP ablation generates a broader zone of direct resistive heating of tissue with a shorter temperature decay time, creating a larger diameter and lesser depth with similar lesion volumes compared with conventional LP ablation (7, 8), which can reduce the risk of steam pops and collateral damage to adjacent structures like the esophagus.

To control and minimize time-dependent deep tissue heat transfer, the ablation duration should be short (2–5 s or no more than 15 s at each location) in the HP setting (9–12). Nevertheless, the subjective determination of each site ablation duration preselected by the operators might lead to incomplete ablation lesions and subsequent increased likelihood of reconnection and gap formation of left atrium-pulmonary vein (LA-PV), which may cause recurrence of AF and atrial tachyarrhythmia/flutter (AT/AFL) (2, 11, 13). The lesion size index (LSI) is a multiparameter index incorporating power, contact force (CF), impedance, and time, and is found to be highly predictive of RF lesion width and depth in *ex vivo* studies, which is a better predictor of RF lesion dimensions than each of its components and is expected to be used as a surrogate end point to determine the duration of ablation (14–16). Recently, it was reported that the HP ablation guided by LSI (LSI-guided HP) could help manage the ablation duration and was shown to be feasible and effective for AF (17, 18). Nevertheless, although several studies have evaluated the optimal LSI cutoff value for predicting acute LA-PV conduction gaps in LP ablation (16, 19), the optimal LSI in HP ablation approach for PVI has yet to be determined. The aim of this study was to explore the efficacy of LSI-guided HP (50 W) ablation technique for PVI and further investigate the association between LSI values and acute conduction gap formation, and further evaluate the optimal LSI in HP ablation for PVI in patients with AF.

## Methods

### Study Population

A total of 105 patients with AF who received LSI-guided HP (50 W) ablation for PVI at Fuwai hospital between June 2019 to July 2020 were consecutively enrolled in this study. Prior to the procedure, the patients were required to take anticoagulant agents for at least 4 weeks. The absence of thrombus in the LA was confirmed using cardiac CT angiogram or transesophageal echocardiogram before the procedure. The key exclusion criteria were 1) prior catheter or surgical ablation for AF; 2) valvular-related AF; 3) LA diameter >55 mm, or left ventricular ejection fraction (LVEF) <35%; 4) stroke, or transient ischemic attack within 6 months; and 5) pregnancy. All demographic and clinical data were extracted in the institutional medical record system. All patients signed informed consent forms, and the study complied with the Declaration of Helsinki and was approved by the Ethics Committee of Fuwai Hospital.

### LSI-Guided HP Ablation for PVI Procedure

The procedure was performed under conscious sedation anesthesia with fentanyl citrate. Local right cervical and groin anesthesia was performed with lidocaine 1%, 5–10 ml. Under fluoroscopy, the decapolar catheter was placed in the coronary sinus by the right internal jugular vein route. After a double trans-septal puncture was performed from right femoral vein access, anticoagulation with heparin was initiated to maintain a target-activated clotting time of 250–350 s. Through transseptal access, a nonsteerable sheath (SL1, 8.5F; Abbott) and a steerable sheath (Agilis, 11.5F; Abbott) were placed into the LA. Then, the 10-pole circular mapping catheter (AFocus II, Abbott) and a CF-sensing catheter with a 3.5-mm tip electrode with six small irrigation holes (TactiCath Quartz; Abbott) were advanced into the LA via the above both sheaths. A three-dimensional electroanatomic mapping system (Ensite V5 system, Abbott) was used to perform an electro-anatomical map of the LA and PVs using the circular mapping catheter.

Contiguous point-by-point ipsilateral PVI for left PVs and right PVs was achieved guided by a three-dimensional mapping system. The decision to perform additional linear ablation depended on the LA substrate and the operator. All ablation lesions were performed using a power-controlled mode with the power limited to 50 W in both the anterior and posterior segments, temperature limit 43°C at 25 ml/min flow rate. The target CF was between 5 and 15 g with target LSI values at 5.0 and 4.0 for anterior and posterior walls, respectively (16). Once the target LSI was reached, the RF application was stopped, and the catheter was moved to an adjacent spot. The ablation duration should not be over 30 s for each ablation point, otherwise reablation was performed after adjusting CF. Surface 12-lead ECG and intracardiac electrograms were recorded continuously at a speed of 100 mm/s on LabSystem Pro (Bard Electrophysiology, Lowell, MA).

### AutoMark Settings

In this study, PVI was conducted with the AutoMark system (Abbott), which automatically detects the ablation duration and calculates force–time integral (FTI) and LSI for each lesion only when the ablation catheter stays within the confined area. FTI was defined as the total CF integrated over the time of RF application. LSI is calculated and displayed in real time that aggregates CF and RF current data across time and is calculated as follows (15):


$$LSI=b_{0}\left(1{-}^{\frac{-F}{b_{1}}}+\;b_{2}\right)\left(1-e^{\frac{-I^{2}}{b_{3}^{2}}}\right)\left(1-b_{4}+\frac{b_{4}\left(1-\frac{-T}{{}^{b_{5}}}\right)}{1{-}^{\frac{-60}{b_{5}}}}\right)$$

where LSI is the lesion index (arbitrary units); b_0−5_ are scaling constants; *F* is a 6-s sliding window average of CF; *I* is a 6-s sliding window average of RF current; and *T* is time.

For catheter position stability, AutoMark settings for filter thresholds were the minimum marker time was 3 s, the marker spacing was 6 mm, and the away time was 5 s. The lesion tag size was 4 mm, and the target interlesion distance (ILD) between the two neighboring lesions was 5 mm or less according to the recommendation of a previous study (20, 21).

### Pulmonary Vein Segments and Ablation Parameters

**Figure 1:**
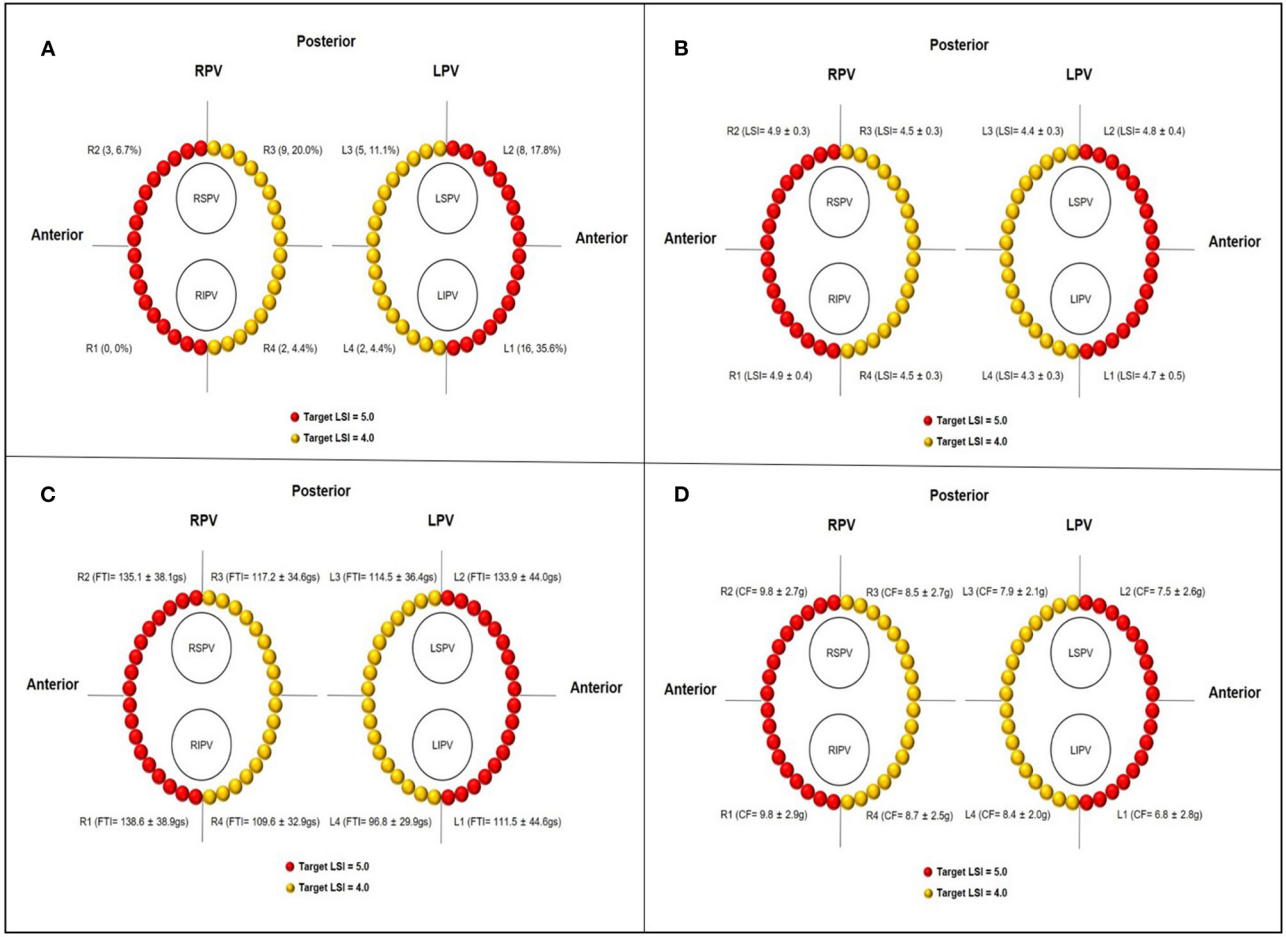
**(A)** Schematic diagrams of target LSI at different PVs segments and distribution of conduction gaps after first-past PVI attempt. **(B–D)** The actual regional LSI, FTI, and CF at different PVs segments, respectively. LPV, left pulmonary vein; RPV, right pulmonary vein; LSPV, left superior pulmonary vein; LIPV, left inferior pulmonary vein; RSPV, right superior pulmonary vein; RIPV, right inferior pulmonary vein; L1 and R1, anterior inferior segments of LPV and RPV, respectively; L2 and R2, anterior superior segments of LPV and RPV, respectively; L3 and R3, posterior superior segments of LPV and RPV, respectively; L4 and R4, posterior inferior segments of LPV and RPV, respectively.

For each ipsilateral pair of PVs, we divided the PV antrum into four regions, including two segments at the anterior wall and two segments at the posterior wall, respectively (as shown in Figure 1A). A total of 210 PV circles (840 PV segments) were analyzed and ablation points were assigned to each segment of the PV antrum. Allowing for a detailed ablation lesion analysis, the following parameters of each ablation site, including FTI, LSI, RF power, CF, RF duration, impedance drop (Δ-Imp), RF temperature, and ILD, were analyzed offline and quantitative measurements for the respective eight circumferential PVs segments were performed in each patient.

### The Definition of Conduction Gaps

PVI was verified as the absence of any PV or LA potential in the PV antral ablation area using a circular catheter and/or the ablation catheter, and bidirectional conduction block between the PV and the LA were also assessed. First-pass PVI was defined if PVI was achieved following complete circumferential PV antral ablation surrounded by a line of contiguous ablation lesions. PVs were further assessed for acute conduction gap formation after a minimum 30-min waiting period of the first-pass completion of ipsilateral circumferential PVs ablation. The location of conduction gap was detected by using the circular catheter and ablation catheter, which was located just adjacent to the ablation line as close as possible, and was defined as a change of clear activation sequence or elimination of PV potential from the LA to PV caused by additional RF application. When one gap site included multiple ablation points with the target tag size, all of these ablation points were counted as gaps. For each subject, the ablation map was carefully reviewed and analyzed offline to identify the conduction gap localization for the respective 8 circumferential PVs segments.

### Statistical Analysis

Continuous data are presented as mean ± SD, and dichotomous data are expressed as numbers and percentages. A comparison of continuous variables between different PVs segments was performed with a one-way analysis of variance (ANOVA) with Bonferroni *post-hoc* testing. A comparison of ablation characteristics with and without conduction gaps was performed using the unpaired samples *t-*test. The univariable and multivariable binary logistic regression analysis used parameters that have already been reported to have a relationship with conduction gaps, and the *p*-values were <0.05 to predict conduction gaps. The predictive value of different threshold levels of ablation parameters for conduction gaps was assessed using sensitivity, specificity, and receiver operating characteristic (ROC) curve analysis. A two-sided *p* < 0.05 was considered statistically significant. All analyses were performed with SPSS for Windows, version 22.0 (SPSS, Chicago, USA).

## Results

### Patient and Procedure Characteristics

The clinical and procedure characteristics at baseline are summarized in Table 1. Of those, 76 (72.4%) were men. The mean age was 57.8 ± 9.8 years, and the mean body mass index was 26.0 ± 3.1 kg/m^2^. The whole study cohort included 59 patients with paroxysmal AF and 46 patients with persistent AF with a mean LA diameter of 39.5 ± 5.5 mm and mean LVEF of 62.0 ± 5.9 %. All patients with targeted PVs (210 ipsilateral veins) were successfully isolated following RF ablation procedure. The first-pass PVI was achieved in 82 (78.1%) patients. The total RF duration for PVI per procedure was 30.4 ± 6.8 min, and the mean fluoroscopy time was 38.0 ± 27.7 s with mean ablation points of 65.6 ± 10.6.

**Table 1:** Baseline clinical and procedure characteristics.

	**Study patients (*n* = 105)**
Age, yrs	57.8 ± 9.8
Male, %	76 (72.4)
BMI, kg/m^2^	26.0 ± 3.1
History of AF, mths	33.8 ± 30.6
CHA_2_DS_2_-VASc score	1.3 ± 1.2
Paroxysmal AF	59 (56.2)
Persistent AF	46 (43.8)
**Complications**	
Hypertension	43 (41.0)
Diabetes mellitus	9 (8.6)
Coronary artery disease	12 (11.4)
Stroke	3 (2.9)
Heart failure	6 (5.7)
**Echocardiography**	0.19 ± 0.49
LAD, mm	39.5 ± 5.5
LVEF, %	62.0 ± 5.9
**Procedure**	
PVI only	67 (63.8)
Ablation points for PVI	65.6 ± 10.6
Ablation duration for PVI, min	30.4 ± 6.8
Fluoroscopy time for PVI, s	38.0 ± 27.7
Additional line ablation	38 (36.2)

*The data are presented as the numbers (%) or the mean ± SD. BMI, body mass index; AF, atrial fibrillation; CHA2DS2-VASc score, congestive heart failure, hypertension, age (≥75 years), diabetes, stroke/transient ischemic attack, vascular disease, age (65-74 years), sex female; LAD, left atrial diameter; LVEF, left ventricular ejection fraction; PVI, pulmonary vein isolation*.

Steam pops without pericardial effusion were found in 3 (0.04%) out of 6,842 lesions and in 3 (2.9%) out of 105 patients, including 2 in the anterior superior segments of right pulmonary vein (RPV) and 1 in the anterior ridge segment of left pulmonary vein (LPV). The mean CF, time and LSI at the site of the steam pops in three patients were 21 g, 6 s, 5.1; 25 g, 5 s, 5.6; 27 g, 6 s, 5.9, respectively. An arteriovenous fistula was found at the puncture site of the right femoral vein in three patients (2.9%) and one patient (1.0%) had a pseudoaneurysm. No esophageal injury, phrenic nerve injury, cardiac tamponade, or stroke occurred.

### Ablation Lesion Analysis

As shown in Figures 1B–D and Table 2, the total number of RF application was 6,842 with 3,269 for the LPV circles and 3,573 for the RPV circles. Overall, the mean LSI value and FTI per lesion were 4.6 ± 0.4 and 120.6 ± 40.4 gs based on ablation duration of 15.4 ± 6.4 s and mean CF of 8.4 ± 2.8 g. The mean Δ-Imp (%) per lesion was 17.3 ± 6.9 Ω (13.8 ± 4.4%), and the mean temperature per lesion was 35.5 ± 2.6°C. The mean ILD between two neighboring lesions was 4.3 ± 0.4 mm. Compared with the ablation lesion parameters of respective left and right posterior segments, left and right anterior segments have significantly higher mean CF and mean temperature, longer ILD and higher LSI (all *p* < 0.05), and tended to have higher FTI, longer ablation duration, and larger mean Δ-Imp.

**Table 2:** Ablation lesion results per segment.

		**Left PV circle**, ***n*** **=** **105**				**Left PV circle**, ***n*** **=** **105**			
		**Left PV lesion**, ***n*** **=** **3269**				**Left PV lesion**, ***n*** **=** **3573**			
	**Overall**	**L1 segment**	**L2 segment**	**L3 segment**	**L4 segment**	**R1 segment**	**R2 segment**	**R3 segment**	**R4 segment**
Lesions, n	6842	966	929	711	663	866	946	878	883
Max CF, g	29.3 ± 14.7	26.6 ± 14.6	22.4 ± 11.9^*^	27.0 ± 13.5	25.9 ± 10.7	30.1 ± 14.2^*^	26.5 ± 10.7^*^	38.5 ± 16.3	36.5 ± 16.3
Min CF, g	1.0 ± 1.5	0.9 ± 1.7^*^	1.2 ± 1.7	1.2 ± 1.5	1.2 ± 1.4	0.9 ± 1.3^*^	1.6 ± 1.7^*^	0.7 ± 1.1	0.8 ± 1.2
Mean CF, g	8.4 ± 2.8	6.8 ± 2.8^*^	7.5 ± 2.6^*^	7.9 ± 2.1	8.4 ± 2.0	9.8 ± 2.9^*^	9.8 ± 2.7^*^	8.5 ± 2.7	8.7 ± 2.5
Max temperature, °C	37.5 ± 3.2	37.4 ± 2.9^*^	39.0 ± 3.2^*^	36.2 ± 2.7	36.0 ± 2.6	39.4 ± 3.1^*^	39.1 ± 3.2^*^	35.7 ± 2.3	36.1 ± 2.6
Min temperature, °C	34.0 ± 2.9	33.8 ± 2.6^*^	35.3 ± 3.0	32.8 ± 2.4	32.8 ± 2.3	35.7 ± 2.9^*^	35.4 ± 2.9^*^	32.6 ± 2.1	32.9 ± 2.3
Mean temperature, °C	35.5 ± 2.6	35.3 ± 2.4^*^	36.6 ± 2.8^*^	34.5 ± 2.2	34.4 ± 2.2	37.0 ± 2.7^*^	36.8 ± 2.7^*^	34.4 ± 2.0	34.6 ± 2.1
Mean Δ-Imp, Ω	17.3 ± 6.9	19.7 ± 8.2^*^	18.3 ± 7.1^*^	17.4 ± 6.4	14.8 ± 5.3	17.4 ± 7.0	16.4 ± 6.9	17.5 ± 6.7	16.3 ± 5.6
Mean Δ-Imp, %	13.8 ± 4.4	15.6 ± 5.1^*^	14.5 ± 4.6^*^	13.6 ± 3.9	12.5 ± 3.6	13.7 ± 4.2	12.9 ± 4.3	13.7 ± 4.1	13.2 ± 3.8
RF duration, s	15.4 ± 6.4	18.1 ± 8.7^*^	19.2 ± 7.8^*^	15.0 ± 4.9	11.8 ± 3.7	15.1 ± 5.4	14.6 ± 5.0	14.7 ± 5.1	13.2 ± 4.5
ILD, mm	4.3 ± 0.4	4.4 ± 0.4^*^	4.4 ± 0.5^*^	4.3 ± 0.4	4.2 ± 0.3	4.3 ± 0.4^*^	4.6 ± 0.4^*^	4.1 ± 0.2	4.2 ± 0.3
FTI, gs	120.6 ± 40.4	111.5 ± 44.6	133.9 ± 44.0^*^	114.5 ± 36.4	96.8 ± 29.9	138.6 ± 38.9^*^	135.1 ± 38.1^*^	117.2 ± 34.6	109.6 ± 32.9
LSI	4.6 ± 0.4	4.7 ± 0.5^*^	4.8 ± 0.4^*^	4.4 ± 0.3	4.3 ± 0.3	4.9 ± 0.4^*^	4.9 ± 0.3^*^	4.5 ± 0.3	4.5 ± 0.3

*The data are presented as the numbers (%) or the mean ± SD. PV, pulmonary vein; CF, contact force; Δ-Imp, impedance drop; ILD, interlesion distance; FTI, force–time integral; LSI, lesion size index*.

* *p < 0.05 (compared with ablation lesion parameters of respective left and right posterior segments). Abbreviations of pulmonary vein segments are as shown in Figure 1*.

### Gaps Distribution

PVs' conduction gaps were detected at 45 (0.7%) points in 7 (87.5%) PV segments in 23 (21.9%) patients. The distribution of gaps within each segment of the PVs is illustrated in Figure 1A. The greatest number of gaps was 16 (35.6%) in the anterior inferior segments of LPV, followed by 9 (20.0%) in the posterior superior segments of RPV and 8 (17.8%) in the anterior ridge segment of LPV, no gaps were found in the anterior inferior segments of RPV. Moreover, gaps were concentrated in the anterior segments of PVs (27, 60.0%), which was significantly more than gaps (18, 40.0%) in the posterior segments of PVs.

### Ablation Parameters With and Without Gaps

The ablation characteristics with and without gaps are shown in Table 3. Although the min CF and mean Δ-Imp were not significantly different between the two groups, the max and mean CF, as well as max, min, and mean temperature, were significantly lower, the ablation duration per RF application was significantly shorter, and the mean ILD was significantly longer in the lesions with gaps than those without gaps. Furthermore, the LSI (3.9 ± 0.4 vs. 4.6 ± 0.4, *p* < 0.001) and FTI (82.6 ± 24.6 vs. 120.9 ± 40.4 gs, *p* < 0.001) were significantly lower in the gap group compared with the nongap group.

**Table 3:** Comparison of ablation lesion characteristics with and without gaps.

	**Without gap**	**With gap**	***P-*value**
	**(*n* = 6797)**	**(*n* = 45)**	
Max CF, g	29.3 ± 14.7	23.5 ± 13.0	0.008
Min CF, g	1.0 ± 1.5	0.8 ± 1.2	0.243
Mean CF, g	8.5 ± 2.8	5.7 ± 2.4	<0.001
Max temperature, °C	37.5 ± 3.2	36.0 ± 1.6	<0.001
Min temperature, °C	34.0 ± 2.9	32.7 ± 1.4	<0.001
Mean temperature, °C	35.6 ± 2.6	34.4 ± 1.4	<0.001
Mean Δ-Imp, Ω	17.3 ± 6.9	18.3 ± 8.5	0.329
Mean Δ-Imp, %	13.8 ± 4.3	13.9 ± 5.3	0.816
RF duration, s	15.4 ± 6.4	10.5 ± 3.6	<0.001
ILD, mm	4.3 ± 0.4	4.4 ± 0.3	0.031
FTI, gs	120.9 ± 40.4	82.6 ± 24.6	<0.001
LSI	4.6 ± 0.4	3.9 ± 0.4	<0.001

*The data are presented as the mean ± SD. CF, contact force; Δ-Imp, impedance drop; ILD, interlesion distance; FTI, force–time integral; LSI, lesion size index*.

For anterior segments, the max, min and mean CF, and temperature were significantly lower, and the ablation duration per lesion was significantly shorter in the gap group compared with the nongap group. For posterior segments, mean CF was significantly lower, the ablation duration per lesion was significantly shorter, and the mean ILD was significantly longer in patients with gaps compared with those without gaps. Not only anterior segments but also posterior segments, both of the LSI and FTI, were significantly lower in the gap group compared with the nongap group (Table 4).

**Table 4:** Comparison of ablation lesion characteristics of anterior and posterior segments with and without gaps.

	**Anterior segments**			**Posterior segments**		
	**Without gap**	**With gap**	***P-*value**	**Without gap**	**With gap**	***P-*value**
	**(*n* = 3680)**	**(*n* = 27)**		**(*n* = 3,117)**	**(*n* = 18)**	
Max CF, g	26.5 ± 13.2	21.4 ± 12.9	0.049	32.7 ± 15.7	26.6 ± 12.8	0.100
Min CF, g	1.1 ± 1.6	0.5 ± 0.9	0.001	0.9 ± 1.3	1.2 ± 1.4	0.298
Mean CF, g	8.5 ± 3.1	4.8 ± 2.0	<0.001	8.4 ± 2.4	7.1 ± 2.5	0.020
Max temperature, °C	38.7 ± 3.2	36.2 ± 1.3	<0.001	36.0 ± 2.5	35.6 ± 1.9	0.437
Min temperature, °C	35.1 ± 2.9	32.9 ± 1.2	<0.001	32.7 ± 2.3	32.3 ± 1.7	0.182
Mean temperature, °C	36.4 ± 2.7	34.6 ± 1.2	<0.001	34.5 ± 2.1	34.1 ± 1.6	0.441
Mean Δ-Imp, Ω	18.0 ± 7.4	18.6 ± 8.7	0.638	16.6 ± 6.1	18.0 ± 8.5	0.324
Mean Δ-Imp, %	14.2 ± 4.7	14.3 ± 5.6	0.908	13.3 ± 3.9	13.4 ± 5.0	0.920
RF duration, s	16.9 ± 7.2	10.0 ± 3.5	<0.001	13.7 ± 4.8	11.2 ± 3.8	0.023
ILD, mm	4.4 ± 0.4	4.4 ± 0.3	0.721	4.2 ± 0.3	4.5 ± 0.3	0.001
FTI, gs	129.8 ± 42.9	86.4 ± 24.6	<0.001	110.3 ± 34.4	77.0 ± 24.3	<0.001
LSI	4.8 ± 0.4	3.9 ± 0.5	<0.001	4.4 ± 0.3	3.9 ± 0.4	<0.001

*The data are presented as the mean ± SD. CF, contact force; Δ-Imp, impedance drop; ILD, interlesion distance; FTI, force–time integral; LSI, lesion size index*.

### Relationships Between Ablation Parameters and Gap Formation

**Figure 2:**
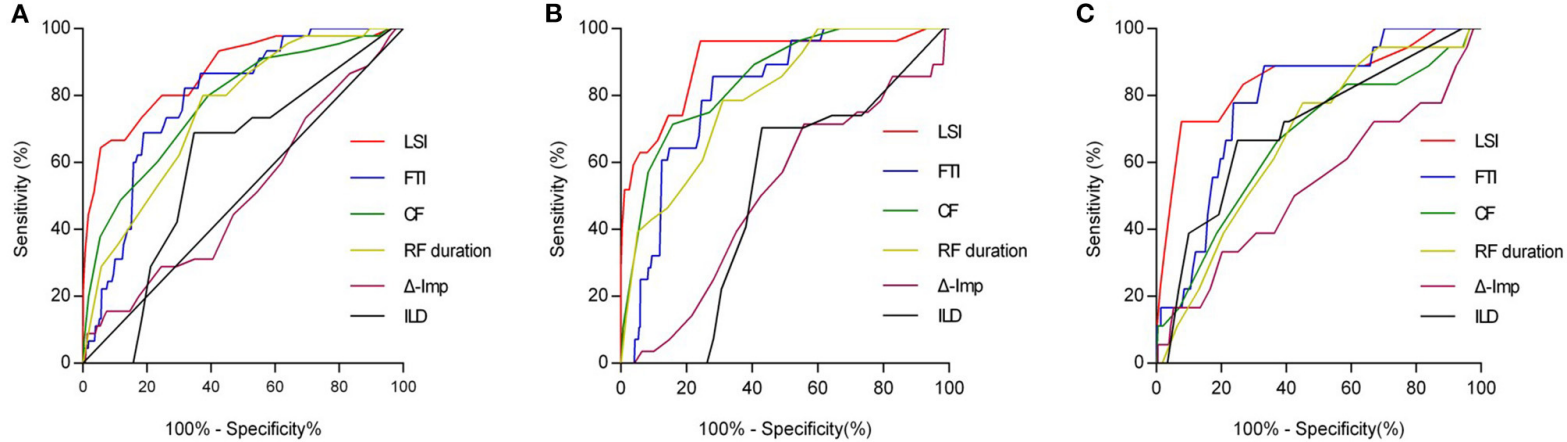
ROC curve analysis for predicting acute conduction gap formation. **(A)** LSI showed the best predictive value with the AUC curve of 0.87 for all PVs segments. AUC values for FTI, CF, RF duration, Δ-Imp, and ILD were 0.79, 0.78, 0.75, 0.51, and 0.60, respectively. The optimal LSI threshold for predicting gap for all PVs segments was 4.35 (sensitivity 80.0%; specificity 75.4%, *p* < 0.0001). **(B)** For anterior wall of PVs, LSI showed the best predictive value with the AUC curve of 0.90. AUC values for FTI, CF, RF duration, Δ-Imp, and ILD were 0.81, 0.85, 0.80, 0.51, and 0.51, respectively. The optimal LSI threshold for anterior wall of PVs was 4.55 (sensitivity 96.3%; specificity 75.8%, *p* < 0.0001). **(C)** For posterior wall of PVs, LSI also showed the best predictive value with the AUC curve of 0.85. AUC values for FTI, CF, RF duration, Δ-Imp, and ILD were 0.79, 0.67, 0.67, 0.53, and 0.71, respectively. The optimal LSI threshold for posterior wall of PVs was 3.95 (sensitivity 72.2%; specificity 92.3%, *p* < 0.0001). ROC, receiver operating characteristic; LSI, lesion size index; AUC, area under the curve; PV, pulmonary vein; FTI, force-time integral; CF, contact force; RF, radiofrequency; Δ-Imp, impedance drop; ILD, interlesion distance.

As shown in Table 5, after adjusting for confounding factors of the significant ablation parameters, the multivariable analysis has shown that LSI was identified as an independent predictor of acute conduction gap formation [odds ratio (OR): 0.62; 95% CI: 0.54 to 0.71, *p* < 0.001). Figure 2A presents the ROC curve analysis for LSI, FTI, CF, RF duration, Δ-Imp, and ILD to determine the thresholds for predicting the presence of acute conduction gap formation. The area under the curve (AUC) values for LSI, FTI, CF, RF duration, Δ-Imp, and ILD were 0.87, 0.79, 0.78, 0.75, 0.51, and 0.60, respectively. Compared to other ablation parameters, LSI showed the best predictive value with an AUC of 0.87 and the cutoff value of LSI on the ROC curve was 4.35 (sensitivity 80.0%; specificity 75.4%, *p* < 0.0001). Hence, the LSI of 4.35 showed the best predictive value for gap formation in all PVs' segments. In addition, following stratification by PVs' segments, the LSI of 4.55 had the highest predictive value for gap formation for the anterior segments (AUC 0.90; sensitivity 96.3%; specificity 75.8%, *p* < 0.0001) and the lower LSI of 3.95 showed a relatively high sensitivity of 72.2% and specificity of 92.3% for posterior segments (AUC 0.85, *p* < 0.0001), respectively, as shown in Figures 2B,C.

**Table 5:** The univariable and multivariable logistic regression analysis for predicting acute conduction gap formation.

	**Univariable**		**Multivariable**	
	**OR (95% CI)**	***P*-value**	**OR (95% CI)**	***P*-value**
LSI	0.58(0.52 - 0.62)	<0.001	0.62(0.54 - 0.71)	<0.001
FTI	0.94(0.93 - 0.95)	<0.001	1.02(0.99 - 1.05)	0.156
Mean CF	0.61(0.53 - 0.70)	<0.001	0.69(0.49 - 0.97)	0.031
RF duration	0.80(0.74 - 0.87)	<0.001	0.82(0.69 - 0.98)	0.028
Mean Δ-Imp	1.02(0.98 - 1.06)	0.328	1.02(0.97 - 1.07)	0.433
ILD	1.79(0.90 - 3.57)	0.095	1.08(0.99 - 1.18)	0.059

*OR, odds ratio; CI, confidence interval; LSI, lesion size index; FTI, force-time integral; CF, contact force; RF, radiofrequency; Δ-Imp, impedance drop; ILD, interlesion distance*.

## Discussion

In this study, we explored the efficacy of LSI-guided HP ablation technique for PVI and further elucidated the relationship between LSI and gap formation, as well as the best cutoff value, to predict gap formation following LSI-guided HP ablation for PVI in a Chinese AF cohort. The most important finding of this study was that LSI-guided HP ablation contributed to isolation of all targeted PVs with a higher first-pass PVI rate. Furthermore, most of the conduction gaps were concentrated in anterior wall while no or few gaps were observed in the posterior wall, and LSI was significantly lower in the gap group compared with the nongap group. In addition, our results showed that LSI turned out to be a strong independent predictor of acute conduction gap formation, and more than 4.35 of LSI for all PVs' segments showed the best predictive value to avoid gap formation for achieving effective first-pass PVI. The optimal LSI of 4.55 for the anterior wall and 3.95 for the posterior wall were the best cutoff values for detecting conduction gaps, respectively.

As is known, long-lasting, continuous, and transmural PVI has the greatest effect on the long-term atrial arrhythmia-free survival, and it is still a clinical challenge (1). Compared to conventional LP ablation, HP ablation distinctly increases resistive heating and decreases conductive heating, avoiding damage depth excessively and thus reducing the risk of adjacent tissue damage, especially esophageal injury (22, 23). Although HP ablation has already acted as a meaningfully efficient and safe strategy for treating AF, it did not significantly reduce the recurrence of AT/AFL compared with conventional LP ablation (24, 25). Recurrent AT/AFL was frequently associated with the reconnection of conduction gaps in the circumferential PVI lines, as well as extrapulmonary areas, following HP ablation (26, 27). Hence, optimization of procedural parameters, including power and duration for HP ablation, is critical for the creation of durable transmural lesions without collateral injury.

LSI is derived from a mathematical expression that incorporates power, CF, impedance, and time, which could predict accurately lesion dimensions by the experimental study and was reported to be related to higher single ablation success rate and lower rate of acute conduction gap formation, subsequently to minimize AT/AF recurrence following PVI (15, 28). Thus, it is important to note that combining the advantage of both HP ablation and LSI may preferably improve the procedural efficacy. Using LSI-guided HP ablation strategy for PVI in our study, despite a relatively low CF of 8.4 ± 2.8 g in our series, all targeted PVs were successfully isolated with a shortening ablation duration of 15.4 ± 6.4 s without severe complications other than steam pop. The first-pass PVI rate was 78.1% in our study, and the incidence of first-pass PVI was reasonably higher and the subsequent incidence of acute conduction gap formation was quite lower when compared to previous LP ablation studies with an average of 61.8% of first-pass PVI (29). The development of tissue edema and subsequent nontransmural lesion, as well as loss of proper tissue CF or catheter dislodgement during prolonged LP ablation, may lead to a lower incidence of first-pass PVI and a higher probability of gap formation. On the contrary, when following an LSI-guided HP ablation strategy, the use of higher power translates into distinctly shorter ablation duration, HP ablation could improve the catheter stability in a short time, achieve transmural injuries by predominant resistive heating, and reduce the conduction gaps, generating a higher first-pass PVI rate (23). Furthermore, in recent POWER-FAST PILOT and PILOT-AF study (17, 18), the first-pass PVI rate was 57% and 73.8% following LSI-guided HP ablation, respectively, which were relatively lower than that in this study. In spite of the similar ablation parameter settings as our study, including energy power output and target LSI, a higher incidence of first-pass PVI in our study may potentially be attributed to the more remarkably shorter RF duration per lesion and better stable tissue contact.

Although the role of LSI in PVI during LP ablation has been well recognized (14, 16, 30), and the optimal LSI in HP ablation to create transmural lesions and avoid conduction gaps remains unclear. In this study, to our knowledge, we are the first to elucidate the relationship between LSI and gap formation, as well as the best cutoff value, to predict gap formation following HP ablation for PVI. Theoretically, increasing LSI values could generate larger lesions and enhance a higher probability of contiguity and transmurality, but bring a higher potential risk of collateral damage (15). It is of great importance to identify the optimal target LSI value providing the best compromise between efficacy and safety. In line with a previous study conducted by Wang et al. (31), we found that more gaps were frequently concentrated in the anterior wall than those in the posterior wall. Moreover, we detected that the LSIs were significantly lower in the gap group compared with the nongap group and low LSI was significantly related to the formation of conduction gap regardless of anterior or posterior segments of PVs. When combined in a multivariable model, LSI represented a strong independent predictor of acute conduction gap formation. On ROC curve analysis, an LSI threshold level of 4.35 was identified to predict gaps in all PVs segments. Considering the wall thickness of the posterior wall of LA thinner than the nonposterior wall, excessive HP ablation of LA posterior wall may result in a rapid rise in tissue temperature and thermal latency to cause overheating of the myocardium and thermal injury to the adjacent tissues (16, 31, 32). Although a recent Frankfurt AI-HP ESO-I/II study demonstrated that the incidences of ablation-related esophageal lesion during HP ablation seem markedly low (33, 34), data from the POWER-FAST PILOT and PILOT-AF studies have shown that esophageal lesions were frequently found in patients with higher LSI when HP ablation on the LA posterior wall (17, 18). Referring to a previous study on LSI settings for ablation on LA posterior wall, minimal RF application was applied to the LA posterior in our study, giving rise to the optimal LSI for the posterior wall with the LSI of 3.95 for detecting conduction gaps, which was lower than the LSI of 4.55 for the anterior wall.

In an *ex vivo* model, when the RF application was delivered under the same LSI, it is worth noting that HP ablation resulted in similar lesion volumes but significantly wider lesion when compared to conventional LP ablation (7, 32). The essential mechanism of different lesion geometries when reaching the same target LSI may be that the HP ablation could quickly produce stronger resistive heating which could create a wider surface lesion area, while conductive heating on the tissue surface was weakened by convective cooling through the blood flow and catheter irrigation flow. Therefore, the larger the lesion surface diameter, the lower is the likelihood of gap formation between lesions in LSI-guided HP ablation. It may explain why a relatively lower LSI threshold under HP ablation could predict gaps in our patients compared with the optimal LSI threshold of 5.25 reported by Kanamori et al. using conventional LP ablation (16, 30). Consequently, our results showed a reduced LSI target value would provide a reasonable approach to LSI-guided HP ablation for PVI, which may improve the procedural efficacy and avoid excessive ablation to minimize the occurrence of complications.

## Limitations

First, this study was a retrospective and single-center study in a relatively small sample size cohort, which was therefore subject to a myriad of biases, particularly selection bias and statistical power limitations. Hence, results from the current data need to be confirmed by further large-scale prospective randomized controlled studies. Second, the procedure in our study was performed under conscious sedation anesthesia rather than deep sedation, and RF ablation may cause discomfort such as chest pain or coughing, body movement, and respiratory instability, which may interfere catheter stability, motion correction reference, the accuracy of three-dimensional electroanatomic mapping, and circumferential PV antral ablation lines. Third, given that the thickness of the PVs antrum is significantly different and LSI does not take into account regional variations in underlying left atrial thickness (31), gap formation therefore may be associated not only with LSI value but also with wall thickness for each ablation point. Further study on the relationship among LSI, wall thickness, and gap formation in the LSI-guided HP ablation is warranted. Fourth, although we analyzed the association between LSI and acute conduction gap formation, the relationship between LSI and redo mapping, as well as long-term AF recurrence, was not performed in our study. The long-term efficacy of LSI-guided HP ablation performed with the optimal LSI settings should be performed and validated in future study. Fifth, as similar results were reported in the previous studies, LSI-guided HP may further minimize the collateral thermal injury (35), whereas the incidence and severity of esophageal injury in this study remain unrevealed due to lack of application of continuous luminal esophageal temperature monitoring. Sixth, the ablation catheter with high irrigation is very efficient at cooling the catheter tip and the adjacent atrial tissue, which may affect the catheter tip temperature, Δ-Imp, and subsequent LSI value (36). Therefore, the results of this study were based on the HP ablation using an ablation catheter with 6 irrigation holes (TactiCath Quartz; Abbott), the optimal LSI value for HP ablation using an ablation catheter with 66 or 56 irrigation holes cannot be derived from our data. Finally, the underlying biophysical and pathophysiological mechanisms of the interaction between LSI and gap formation in different PVs segments following HP ablation are also needed to elucidate in further *in vivo* and *ex vivo* studies.

## Conclusion

This study on LSI-guided HP ablation for PVI demonstrated that LSI was correlated with gap formation at different PVs segments and could be utilized as a surrogate end point to guide PVI. To achieve a higher first-pass PVI without acute conduction gaps, more than 4.35 of LSI for all PVs segments showed the best predictive value to avoid gap formation. In addition, the optimal LSI of 4.55 for the anterior segments and 3.95 for the posterior segments of PVs were the best cutoff values for predicting gap formation in LSI-guided HP ablation, respectively.

## Data Availability Statement

The original contributions presented in the study are included in the article/supplementary material, further inquiries can be directed to the corresponding authors.

## Ethics Statement

The studies involving human participants were reviewed and approved by the Ethics Committee of Fuwai Hospital. The patients/participants provided their written informed consent to participate in this study.

## Author Contributions

JW provided the design of the study, analyzed and interpreted data, drafted the manuscript, and approved the final version of the manuscript. CC participated in drafting the manuscript, analyzing and interpreting data. J-MC and YY assisted with the revising of the article. H-XN, WH, and SZ contributed to acquiring the patients' clinical data. All authors contributed to the article and approved the submitted version.

## Conflict of Interest

The authors declare that the research was conducted in the absence of any commercial or financial relationships that could be construed as a potential conflict of interest.

## Publisher's Note

All claims expressed in this article are solely those of the authors and do not necessarily represent those of their affiliated organizations, or those of the publisher, the editors and the reviewers. Any product that may be evaluated in this article, or claim that may be made by its manufacturer, is not guaranteed or endorsed by the publisher.

## References

[1] January CT Wann LS Calkins H Chen LY Cigarroa JE Cleveland JJ. 2019 AHA/ACC/HRS Focused Update of the 2014 AHA/ACC/HRS guideline for the management of patients with atrial fibrillation: a report of the American College of Cardiology/American Heart Association Task Force on Clinical Practice Guidelines and the Heart Rhythm Society in Collaboration With the Society of Thoracic Surgeons. Circulation. (2019) 140:e125–51. 10.1161/CIR.0000000000000665 30686041

[2] Kottmaier M Popa M Bourier F Reents T Cifuentes J Semmler V. Safety and outcome of very high-power short-duration ablation using 70 W for pulmonary vein isolation in patients with paroxysmal atrial fibrillation. Europace. (2020) 22:388–93. 10.1093/europace/euz342 31872249

[3] Chen S Schmidt B Bordignon S Tohoku S Urban VC Schulte Hahn B. Catheter ablation of atrial fibrillation using ablation index-guided high-power technique: Frankfurt AI high-power 15-month follow-up. J Cardiovasc Electrophysiol. (2021) 32:616–24. 10.1111/jce.14912 33484215

[4] Brien J Obeidat M Kozhuharov N Ding WY Tovmassian L Bierme C. Procedural efficiencies, lesion metrics, and 12-month clinical outcomes for Ablation Index-guided 50 W ablation for atrial fibrillation. EP Europace. (2021) 23:878–86. 10.1093/europace/euab031 33693677

[5] Naniwadekar A Dukkipati SR. High-power short-duration ablation of atrial fibrillation: a contemporary review. Pacing Clin Electrophysiol. (2021) 44:528–40. 10.1111/pace.14167 33438279

[6] Park JW Yang SY Kim M Yu HT Kim TH Uhm JS. Efficacy and safety of high-power short-duration radiofrequency catheter ablation of atrial fibrillation. Front Cardiovasc Med. (2021) 8:709585. 10.3389/fcvm.2021.709585 34692779 PMC8530188

[7] Bourier F Duchateau J Vlachos K Lam A Martin CA Takigawa M. High-power short-duration versus standard radiofrequency ablation: Insights on lesion metrics. J Cardiovasc Electrophysiol. (2018) 29:1570–5. 10.1111/jce.13724 30168230

[8] Borne RT Sauer WH Zipse MM Zheng L Tzou W Nguyen DT. Longer duration versus increasing power during radiofrequency ablation yields different ablation lesion characteristics. JACC Clin Electrophysiol. (2018) 4:902–8. 10.1016/j.jacep.2018.03.020 30025690

[9] Reddy VY Grimaldi M De Potter T Vijgen JM Bulava A Duytschaever MF. Pulmonary vein isolation with very high power, short duration, temperature-controlled lesions: the QDOT-FAST trial. JACC Clin Electrophysiol. (2019) 5:778–86. 10.1016/j.jacep.2019.04.009 31320006

[10] Bunch TJ Day JD. Novel ablative approach for atrial fibrillation to decrease risk of esophageal injury. Heart Rhythm. (2008) 5:624–7. 10.1016/j.hrthm.2007.11.007 18325845

[11] Bunch TJ May HT Bair TL Crandall BG Cutler MJ Mallender C. Long-term outcomes after low power, slower movement versus high power, faster movement irrigated-tip catheter ablation for atrial fibrillation. Heart Rhythm. (2020) 17:184–9. 10.1016/j.hrthm.2019.08.001 31398477

[12] Mao ZJ Pei Y Lin H Xiang Y Huang ZQ Xiao FY. Assessment of high-power catheter ablation in patients with atrial fibrillation: a meta-analysis. Front Cardiovasc Med. (2021) 8:609590. 10.3389/fcvm.2021.609590 34746245 PMC8564349

[13] Berte B Hilfiker G Russi I Moccetti F Cuculi F Toggweiler S. Pulmonary vein isolation using a higher power shorter duration CLOSE protocol with a surround flow ablation catheter. J Cardiovasc Electrophysiol. (2019) 30:2199–204. 10.1111/jce.14122 31424123

[14] Mattia LD Crosato M Indiani S Causin E Licciardello C Maria Squasi PA. Prospective evaluation of lesion index-guided pulmonary vein isolation technique in patients with paroxysmal atrial fibrillation: 1-year follow-up. J Atrial Fibrillat. (2018) 10:1858. 10.4022/jafib.1858 29988268 PMC6009787

[15] Calzolari V De Mattia L Indiani S Crosato M Furlanetto A Licciardello C. *In vitro* validation of the lesion size index to predict lesion width and depth after irrigated radiofrequency ablation in a porcine model. JACC Clin Electrophysiol. (2017) 3:1126–35. 10.1016/j.jacep.2017.08.016 29759495

[16] Kanamori N Kato T Sakagami S Saeki T Kato C Kawai K. Optimal lesion size index to prevent conduction gap during pulmonary vein isolation. J Cardiovasc Electrophysiol. (2018) 29:1616–23. 10.1111/jce.13727 30176083

[17] Leo M Pedersen M Rajappan K Ginks MR Hunter RJ Bowers R. Power, lesion size index and oesophageal temperature alerts during atrial fibrillation ablation. Circ Circ Arrhythm Electrophysiol. (2020) 13:e008316. 10.1161/CIRCEP.120.008316 32898435

[18] Castrejón-Castrejón S Martínez Cossiani M Ortega Molina M Escobar C Froilán Torres C Gonzalo Bada N. Feasibility and safety of pulmonary vein isolation by high-power short-duration radiofrequency application: short-term results of the POWER-FAST PILOT study. J Interv Card Electr. (2020) 57:57–65. 10.1007/s10840-019-00645-5 31713704

[19] Dello RA Fassini GM Casella M Romanelli E Pala S Riva S. Lesion index: a novel guide in the path of successful pulmonary vein isolation. J Interv Card Electrophysiol. (2019) 55:27–34. 10.1007/s10840-018-0487-z 30515625

[20] El HM Taghji P Phlips T Wolf M Demolder A Choudhury R. Determinants of acute and late pulmonary vein reconnection in contact force-guided pulmonary vein isolation: identifying the weakest link in the Ablation Chain. Circ Arrhythm Electrophysiol. (2017) 10:e004867. 10.1161/CIRCEP.116.004867 28381417

[21] Park CI Lehrmann H Keyl C Weber R Schiebeling J Allgeier J. Mechanisms of pulmonary vein reconnection after radiofrequency ablation of atrial fibrillation: the deterministic role of contact force and interlesion distance. J Cardiovasc Electrophysiol. (2014) 25:701–8. 10.1111/jce.12396 24575734

[22] Thiyagarajah A Kadhim K Lau DH Emami M Linz D Khokhar K. Feasibility, safety, and efficacy of posterior wall isolation during atrial fibrillation ablation: a systematic review and meta-analysis. Circ Arrhythm Electrophysiol. (2019) 12:e7005. 10.1161/CIRCEP.118.007005 31401853

[23] Leshem E Zilberman I Tschabrunn CM Barkagan M Contreras-Valdes FM Govari A. High-power and short-duration ablation for pulmonary vein isolation. JACC Clin Electrophysiol. (2018) 4:467–79. 10.1016/j.jacep.2017.11.018 30067486

[24] Shin DG Ahn J Han SJ Lim HE. Efficacy of high-power and short-duration ablation in patients with atrial fibrillation: a prospective randomized controlled trial. Europace. (2020) 22:1495–501. 10.1093/europace/euaa144 32810203

[25] Sun X Qi P Yang B Li Z Bie Z Li X. The procedural efficiency, efficacy and safety of high power and short duration ablation in patients with atrial fibrillation: a systemic review and meta-analysis. Int J Cardiol. (2021) 325:76–81. 10.1016/j.ijcard.2020.10.030 33080286

[26] Kuck KH Hoffmann BA Ernst S Wegscheider K Treszl A Metzner A. Impact of complete versus incomplete circumferential lines around the pulmonary veins during catheter ablation of paroxysmal atrial fibrillation: results from the gap-atrial fibrillation-german atrial fibrillation competence network 1 trial. Circ Arrhythm Electrophysiol. (2016) 9:e3337. 10.1161/CIRCEP.115.003337 26763226

[27] Mohanty S Trivedi C Della Rocca DG Gianni C MacDonald B Quintero Mayedo A. Recovery of conduction following high-power short-duration ablation in patients with atrial fibrillation: a single-center experience. Circ Arrhythm Electrophysiol. (2021) 14:e010096. 10.1161/CIRCEP.121.010096 34583523

[28] Taghji P El HM Phlips T Wolf M Knecht S Vandekerckhove Y. Evaluation of a strategy aiming to enclose the pulmonary veins with contiguous and optimized radiofrequency lesions in paroxysmal atrial fibrillation: a pilot study. JACC Clin Electrophysiol. (2018) 4:99–108. 10.1016/j.jacep.2017.06.023 29600792

[29] Winkle RA. HPSD ablation for AF high-power short-duration RF ablation for atrial fibrillation: a review. J Cardiovasc Electrophysiol. (2021) 32:2813–23. 10.1111/jce.14863 33382506

[30] Liu X Gui C Wen W He Y Dai W Zhong G. Safety and efficacy of high power shorter duration ablation guided by ablation index or lesion size index in atrial fibrillation ablation: a systematic review and meta-analysis. J Interv Cardiol. (2021) 2021:1–12. 10.1155/2021/5591590 34149322 PMC8192211

[31] Wang Y Zhou G Chen S Wei Y Lu X Xu J. Tailored ablation index for pulmonary vein isolation according to wall thickness within the ablation circle. Pacing Clin Electrophysiol. (2021) 44:575–85. 10.1111/pace.14125 33184894

[32] Takemoto M Takami M Fukuzawa K Kiuchi K Kurose J Suehiro H. Different tissue thermodynamics between the 40 W and 20 W radiofrequency power settings under the same ablation index/lesion size index. J Cardiovasc Electrophysiol. (2019) 31:196–204. 10.1111/jce.14285 31750592

[33] Chen S Schmidt B Seeger A Bordignon S Tohoku S Willems F. Catheter ablation of atrial fibrillation using ablation index–guided high power (50 W) for pulmonary vein isolation with or without esophageal temperature probe (the AI-HP ESO II). Heart Rhythm. (2020) 17:1833–40. 10.1016/j.hrthm.2020.05.029 32470628

[34] Chen S Chun K Tohoku S Bordignon S Urbanek L Willems F. Esophageal endoscopy after catheter ablation of atrial fibrillation using ablation-index guided high-power: Frankfurt AI-HP ESO-I. JACC Clin Electrophysiol. (2020) 6:1253–61. 10.1016/j.jacep.2020.05.022 33092751

[35] Winkle RA Mohanty S Patrawala RA Mead RH Kong MH Engel G. Low complication rates using high power (45–50 W) for short duration for atrial fibrillation ablations. Heart Rhythm. (2019) 16:165–9. 10.1016/j.hrthm.2018.11.031 30712645

[36] Ullah W Hunter RJ Finlay MC McLean A Dhinoja MB Sporton S. Ablation index and surround flow catheter irrigation: impedance-based appraisal in clinical ablation. JACC Clin Electrophysiol. (2017) 3:1080–8. 10.1016/j.jacep.2017.03.01 29759489

